# Polar metal phase stabilized in strained La-doped BaTiO_3_films

**DOI:** 10.1038/s41598-017-04635-3

**Published:** 2017-07-05

**Authors:** K. S. Takahashi, Y. Matsubara, M. S. Bahramy, N. Ogawa, D. Hashizume, Y. Tokura, M. Kawasaki

**Affiliations:** 1 RIKEN Center for Emergent Matter Science (CEMS), Wako, 351-0198 Japan; 2 PRESTO, Japan Science and Technology Agency (JST), Chiyoda-ku, Tokyo 102-0075 Japan; 3 Institute for Materials Research, Tohoku University, Sendai, 908-8577 Japan; 4 Department of Applied Physics and Quantum Phase Electronics Center, University of Tokyo, Tokyo, 113-8656 Japan

## Abstract

Ferroelectric polarization and metallic conduction are two seemingly irreconcilable properties that cannot normally coexist in a single system, as the latter tends to screen the former. Polar metals, however, defy this rule and have thus attracted considerable attention as a new class of ferroelectrics exhibiting novel properties. Here, we fabricate a new polar metal film based on the typical ferroelectric material BaTiO_3_by combining chemical doping and epitaxial strain induced by a substrate. The temperature dependences of the *c*-axis lattice constant and the second harmonic generation intensity of La-doped BaTiO_3_films indicate the existence of polar transitions. In addition, through La doping, films become metallic at the polar phase, and metallicity enhancement at the polar state occurs in low-La-doped films. This intriguing behaviour is effectively explained by our first-principles calculations. Our demonstration suggests that the carrier doping to ferroelectric material with epitaxial strain serves as a new way to explore polar metals.

## Introduction

The coexistence of two seemingly incompatible states in materials such as multiferroics (showing ferromagnetic and ferroelectric order)^1, 2^and magnetic superconductors^3, 4^has attracted considerable interest. In particular, metallic conduction in the ferroelectric state is an interesting subject. Although Anderson and Blount proposed in the 1960s that a continuous structural phase transition is possible, even in a polar metal^5^, it is rare to see such a transition^6–8^. In addition to such contradictions between polar and metallic states, it is interesting to note the non-centrosymmetric structure itself in solids. For example, a giant Rashba-type split in a non-centrosymmetric structure with a strong atomic spin-orbit interaction discovered in semiconductor BiTeI^9^and ferroelectric-like soft phonons is predicted to stabilize a superconducting phase with a non-centrosymmetric ordering in BiS_2_^10^. Barium titanate BaTiO_3_(BTO) is best known as a typical ferroelectric material presenting high levels of ferroelectric polarization and a weak coercive field^11–14^. Over the past decade, electron-doped BaTiO_3-*δ*_bulk single crystals have been studied to understand the relationship between the crystal structure and transport properties. These studies have revealed the coexistence of a ferroelectric-like lattice distortion and metallic phase^15–17^. However, transport properties have been found to be strongly affected by twin boundaries formed through structural phase transitions towards lower symmetries. In addition, neutron diffraction studies postulate the existence of phase separation between the ferroelectric insulating tetragonal phase and the paraelectric metallic cubic phase^18^. For thin films, electron doped epitaxial BaTi_1-x_Nb_x_O_3-y_films have been grown to reveal insulator-metal transitions^19, 20^. The films were insulated for *x*≤ 0.05 and turned metallic at above *x* ~ 0.2, which is far above the doping level of the present study. By means of electrostatic carrier doping, BTO single crystalline films^21^and SmTiO_3_/BTO heterostructures^22^have been shown to be conductive. However, the relationship between carrier doping and polarity in BTO remains elusive. Therefore, it is unclear and under debate whether a polar metal phase is realizable in an electron-doped BTO.

Here, we show that thin films of electron-doped BTO can indeed enter a polar metal phase. We grew La-doped BTO (La-BTO) films by substituting Ba^2+^with La^3+^for electron doping. Metalorganic gas-source (MO) molecular beam epitaxy (MBE) was used for film growth. This method has proven effective for growing high-quality complex oxide thin films^21, 23–25^. Films grown in this way are free of twin boundaries due to a coherent epitaxy with a biaxial compressive strain from a substrate. We investigated systematic variations in the structural phase transition via X-ray diffraction (XRD) and using optical second harmonic generation (SHG) measurements. The temperature dependences of XRD and SHG indicate that the transitional temperature from non-polar to polar states decreases as La doping increases. The La-BTO film exhibits metallic conduction at an electron density of 10^20^–10^21^ cm^−3^. Interestingly, the slope of the resistivity curve *ρ*(*T*) changes at the transition temperature for low-doped films, implying that metallicity enhances at the polar state rather than at the non-polar state. We found that a characteristic change in the band structure occurs during phase transition under a low doping regime via first-principles calculations, resulting in the enhancement of metallicity.

## Results

### Crystal structure of La-doped BaTiO_3_films

**Figure 1:**
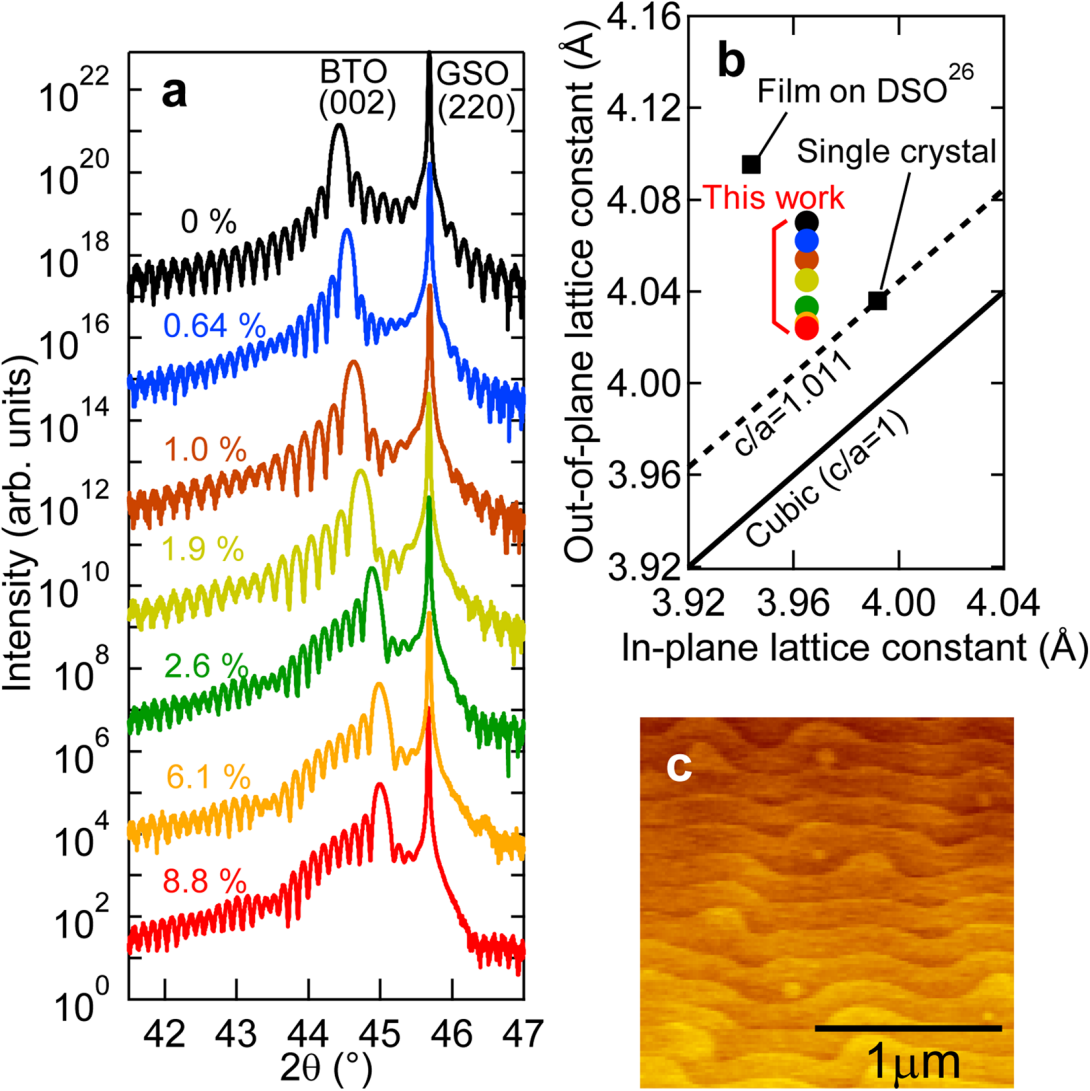
Crystal structure of La-doped BaTiO_3_films. (**a**) 2*θ* − *θ*X-ray diffraction pattern of non-doped BTO and La-BTO films grown on GSO (110) substrates for the (002) peak of BTO and the (220) peak of GSO. (**b**) Lattice constants for non-doped BTO and La-BTO films, bulk single-crystalline BTO, BTO films on DSO and GSO substrates grown by MBE^26^. The solid line denotes the cubic structure (*c*/*a* = 1) and the dotted line denotes the tetragonal structure (*c*/*a* = 1.011) corresponding to single-crystalline BTO. (**c**) AFM image (2 × 2 μm^2^) of the surface of the BTO film.

We grew single-crystalline La-BTO films on GdScO_3_(GSO) (110) substrates via MOMBE (see Methods). An atomic force microscopy (AFM) image of the film is shown in Fig. 1c, whereby the film exhibits a step-and-terrace structure. The step height is approximately 4 Å, corresponding to the height of a single BTO unit cell. The *a*- and *c*-axes lattice constants of the ferroelectric tetragonal phase BTO at room temperature are 3.992 and 4.036 Å, respectively. The lattice constants of orthorhombic GSO are *a* = 5.488 Å, *b* = 5.746 Å, and *c* = 7.934 Å. The mismatch of the in-plane lattice between the GSO (110) substrate (3.970 Å) and *c*-axis oriented BTO is calculated as +0.55%. Choi *et al*. reported that the ferroelectric tetragonal phase is stabilized in a compressively strained BTO film on a GSO substrate, resulting in the enhancement of its Curie temperature (*T*_C_) from 410 K for bulk to 673 K^26^. Figure 1ashows 2*θ* − *θ*scan around (220) peak of GSO substrates for La-BTO films grown in this manner. The La concentration is defined as [*n*]/Ti or as the ratio of the charge carrier density [*n*] at 300 K deduced by Hall effect measurements to the titanium atomic density (see Supplementary Note 1). Sharp (002) film peaks and clear Laue’s fringes can be seen for all the films, indicating that the crystalline quality of La-BTO is comparable to that of non-doped BTO. The thicknesses of La-doped layers are deduced as 42–60 nm from the Laue’s fringes. Reciprocal space mapping results of XRD measurements indicate that the in-plane lattice constant is locked to that of the substrate (not shown). Grown on GdScO_3_with a smaller in-plane lattice (*a* = 3.970 Å) than that of bulk BTO, the films are compressively strained and the out-of-plane lattice (*c*) is elongated. Figure 1bshows the relations of *c*and *a*mapped for La-BTO films in this work together with those of bulk single-crystalline BTO^27, 28^and a BTO film on DSO and GSO substrates^26^. Tetragonality *c*/*a*is known to be an important parameter of the ferroelectric properties of BTO such as *T*_C_and ferroelectric polarization^26^. The tetragonality of La-BTO films on GSO falls between those of single crystal (*c*/*a* = 1.011) in the ferroelectric tetragonal phase and a BTO film on DSO (in-plane lattice constant: 3.944 Å) (*c*/*a* = 1.038).

**Figure 2:**
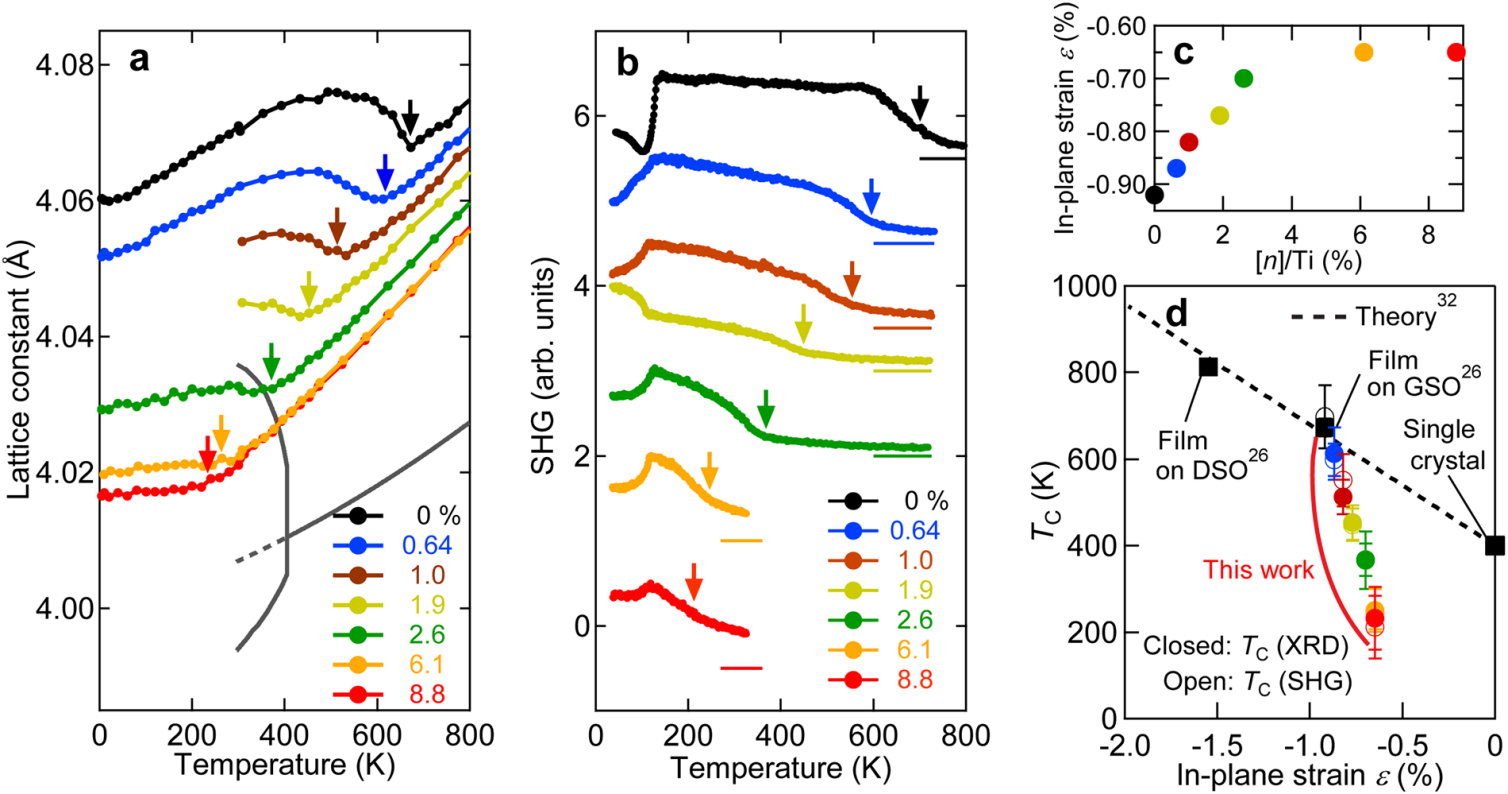
Polar transitions of La-doped BaTiO_3_films. (**a**) Temperature dependence of the *c*-axis lattice constant for non-doped BTO and La-BTO films grown on GSO (110) together with that of lattice constants for the cubic and tetragonal phases of BTO single crystal^26, 27^. The dotted curve denotes the extrapolated lattice constant of BTO single crystals in the cubic phase. (**b**) Temperature dependence of the optical SHG intensity of the films. (**c**) La doping ([*n*]/Ti) dependence of in-plane strain *ε*. (**d**) Non-polar to polar transition temperature *T*_C_as a function of the in-plane strain *ε*of non-doped and La-doped BTO films on GSO, of non-doped BTO films on GSO and of DSO^26^with a theoretical line based on the phase-field simulation^32^.

Figure 2ashows the temperature dependence of the *c*–axis lattice constant for various La-BTO films and lattice constants for the cubic and tetragonal phases of bulk single crystals^27, 28^. Clear anomalies observed in all of the La-BTO films are denoted by arrows. The anomalies monotonically shift to lower temperatures with increasing La doping to up to 8.8%. The anomalies observed from 0.64 to 8.8% doped films reflect the ferroelectric transition in non-doped BTO. As noted below in the section on transport properties, the polarization direction of doped films cannot be reversed by an electric field due to screening by doped mobile electrons. Thus, such doped films should not be defined as ferroelectric but as polar materials at low temperature phases. This result therefore highlights the existence of a low temperature ‘polar phase’ in the La-BTO films. To rule out the coexistence of two phases around the anomaly, we show in Supplementary Fig. 2the temperature dependence of XRD data whereby the film peak has a similar intensity and peak width across the transition. This result is in clear contrast to those of the neutron diffraction data, which indicate phase coexistence^18^. The temperature dependence of second harmonic generation (SHG) intensity is shown in Fig. 2b(See Methods). For each curve, the intensity is normalized against the maximum value as 1 and curves are vertically offset as denoted by horizontal bars. In all of the curves, transitions to an SHG active state are observed at temperatures denoted by the arrows. Although the appearance of SHG is an insufficient condition for ferroelectricity or polar states, the onset temperatures almost coincide with the *T*_C_values determined by the temperature dependence of the *c*-axis lattice constant shown in Fig. 2afor all of the films. Such a coincidence implies that the strong SHG signal is attributable to the polar structure. All the films also show an abrupt change in their intensities at approximately 130 K. As this temperature is independent of La doping, the corresponding change must be related to crystal or surface structure transitions of the GSO substrate. Future studies are required to verify the causes of this behaviour.

At the high temperature paraelectric phase, the lattice constant systematically decreases as La doping levels increase, resulting in lower levels of compressive strain on the La-doped films. Note that such a suppression of *T*_C_is much stronger than that caused by disorder effects arising from A-site doping^29–31^. One can expect the lowering of *T*_C_by La doping results from two effects: a strain effect (the compressive strain decreases with La doping) and the electron carrier doping effect. For the latter, the cause is obvious; mobile carriers destabilize the polar state. Here, to estimate the strain effect, we assume that Poisson’s ratio is the same for non-doped and La-doped films at 0.33 (details are explained in Supplementary Note 2). Figure 2cshows the estimated in-plane strain *ε*of the films as a function of [*n*]/Ti (a definition of [*n*]/Ti is presented in Supplementary Note 1). In Fig. 2d, the transition temperature to a polar state as a function of *ε*is shown for films presented in this work, for the non-doped BTO film on the GSO and DSO^26^﻿and bulk single crystal, and for the theoretical line of strain effects for non-doped BTO based on a phase-field simulation^32^. Although the *T*_C_values of non-doped BTO on GSO and DSO (black symbols) fit the theoretical line (broken line), it is clear that the *T*_C_values of La-BTO films are lower than the theoretical line and that the deviation between the observed *T*_C_for La-BTO films and the theoretical value increases as La doping increases. Therefore, we expect that the *T*_C_is determined and reduced primarily by carrier doping effects in addition to in-plane strain.

### Transport properties of La-doped BaTiO_3_films

**Figure 3:**
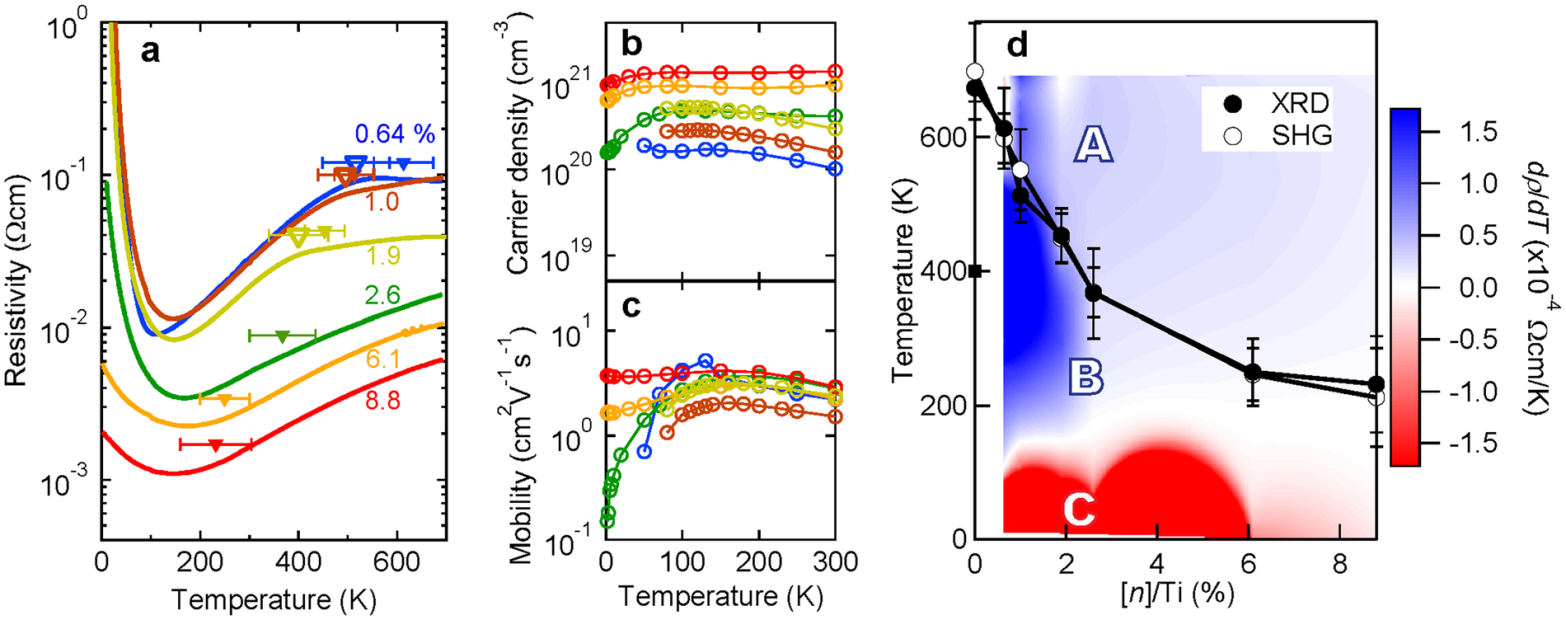
Transport properties of La-doped BaTiO_3_films. Temperature dependence of (**a**) resistivity, (**b**) carrier density, and (**c**) mobility for La-BTO films from [*n*]/Ti = 0.64% to 8.8%. Closed triangles in (**a**) denote the transition temperature from the non-polar phase to the polar phase determined by XRD and open triangles denote the kink temperature where the slope of resistivity as a function of *T*changes. (**d**) Phase diagram of the films. Non-polar to polar transition temperatures determined by XRD shown as closed circles and by SHG shown as open circles are plotted with the counter plot of differentiation of *ρ*(*Τ*), *dρ*/*dΤ*. A phase: paraelectric metal, B phase: polar metal, and C phase: polar insulator are indicated.

For transport measurements, the samples were cut into 5 mm × 1 mm pieces and aluminium wire was ultrasonically bonded to ensure ohmic contact. Figure 3shows the temperature dependence of (a) resistivity, (b) carrier density and (c) mobility for the doped films. As La doping increases, resistivity systematically decreases and carrier density increases. The activation ratio between the La amount measured via quartz crystal microbalance and the measured carrier density is approximately 50–100%. From the data collected at room temperature shown in Fig. 3b, [n]/Ti for each film is determined. Therefore, the [*n*]/Ti is not the actual La density but is an effective electron carrier density (see Supplementary Note 1). As shown in Fig. 3a, surprisingly, the very low carrier doped film (0.64%; *n* = 1.0 × 10^20^ cm^−3^) presents metallic behaviour down to 100 K. It is known that SrTiO_3_exhibits metallic behaviour at much lower doping levels: 0.001–0.01%. The difference of threshold doping level for metallic behaviour can be explained by the strength of electron-phonon coupling. In BTO, small polarons are formed due to the strong electron-phonon coupling while such a coupling is weak in the case of STO leading to the formation of large polarons which cause an overlapping of the trapped potential and the band conduction in a low doping concentration^, 33, 34^. Although optical measurements suggest that longitudinal-optical phonon and soft transverse-optical phonon might contribute to the strong coupling in BTO, the origin of the different nature of the electron-phonon couplings is still not clear. It is, however, notable that previous works^19, 20^have reported threshold conductivity in BTO at a much higher doping level (20%) than that reported here. We consider two reasons for such persistent metallic behaviour at low doping levels. First, the high crystallinity, which is achieved by MOMBE growth excludes carrier trapping states caused by impurities and defects. Second, an absence of twin grain boundaries can lead to the same effect. As the tetragonal structure is stabilized by the epitaxial compressive strain of a substrate as noted above and as any thermal hysteresis behaviours due to structure transitions cannot be observed, films do not suffer from twin boundaries like those found in bulk crystals^15, 16^. Such twin boundaries are known to be a source of trapping sites. The closed triangles presented in Fig. 2ashow each polar transition temperature determined by the XRD measurement. Interestingly, for low-doped films (0.64, 1.0, and 1.9%), each polar transition temperature almost coincides with the kink temperature (denoted by open triangles), which corresponds to the temperature at which the slope of resistivity as a function of *T*changes. Such a change indicates that metallicity is enhanced at a polar state rather than at a non-polar state, as is discussed below in detail. The temperature dependence shown in Fig. 3aindicates that our doped BTO samples are not behaving like a conventional metal. In low temperature regime up to 150 K, the resistivity tends to decrease by increasing the temperature. This is a typical behavior expected in doped semiconductors due to the thermal excitation of trapped carriers. Such an increase of mobility due to the thermal activation of carriers has been discussed in the context of small polaron theories and is ascribed to the strong coupling of charge carriers with high energy longitudinal optical phonons^33, 35, 36^. Such excitations appear to reach a saturating point at 150 K. Accordingly at higher temperatures, our BTO samples behave differently, manifested by a monotonic increase of resistivity. Like a normal metal, this behavior is expected to be due to the suppression of mobility. As such, we don’t regard the low temperature phase as a polar metal phase but rather a polar insulator phase as shown in Fig. 3d. Further investigation to model the conduction mechanism is needed. Figure 3dpresents a phase diagram of films determined by XRD and SHG experiments as a function of [*n*]/Ti. In addition, we superimpose the *dρ*/*dΤ*data to clearly understand the relationship between transport properties and crystal phase transition. *T*_C_determined by the slope change of the *c*-axis lattice constant presented in Fig. 2ais plotted as a closed circle and that determined by the onset temperature of SHG intensity presented in Fig. 2bis shown as an open circle. The ferroelectric transition temperature of bulk BTO is also plotted as a closed square for comparisons at [*n*]/Ti = 0. The non-polar paraelectric region is denoted as ‘A’. The polar metal phase is denoted as ‘B’ whereas the polar insulator phase is denoted as ‘C’. Interestingly, one can find a broad polar metal phase region in the phase diagram of La-doped BTO film. Although the non-polar to polar transition temperature dramatically decreases with La doping, a broad polar metal phase remains due to an enhancement of the transition temperature induced by compressive strain.

### Electronic structures of La-doped BaTiO_3_

To develop an in-depth understanding of the origins of such an intriguing polar metal phase in the epitaxially strained BTO film, we performed a set of relativistic density functional theory calculations for bulk La-doped BTO using the full-potential augmented plane-wave method and the Perdew-Burke-Ernzerhof exchange-correlation function modified by the Becke-Johnson potential via the WIEN2K program^37^. The effect of La doping is treated through a virtual crystal approximation. At each La concentration *x*, corresponding experimental values of lattice parameters *c*and *a*are taken, whereas ionic positions are allowed to be fully optimized until the magnitude of force on each ion becomes less than 0.5 mRy/Bohr. The Brillouin zone is sampled using a 10 × 10 × 10 *k*-mesh.

**Figure 4:**
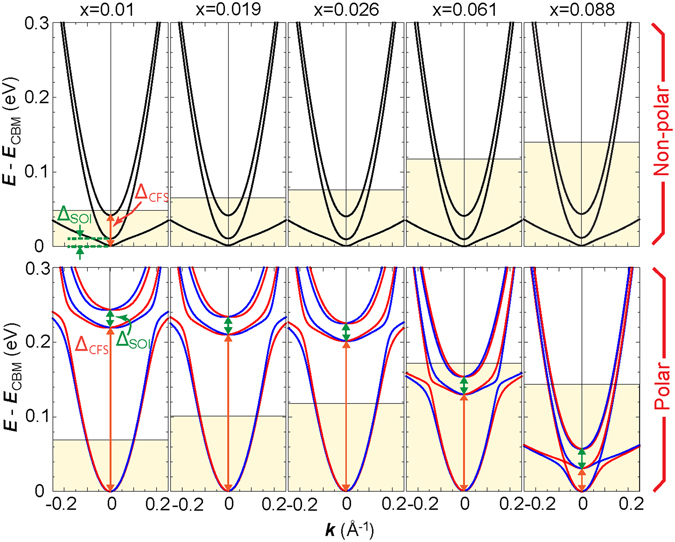
Electronic structures of La-doped BaTiO_3_films. Band structure of La-doped BTO calculated at various La concentrations, *x*, for the non-polar (polar) structure in the upper (lower) panel. The energy separations of bands caused by crystal field splitting and spin orbit interactions are by Δ_CFS_and Δ_SOI_, respectively. For the polar cases, red and blue colours represent the opposite spin states of the bands (red: spins pointing out of the page; blue: spins pointing inward). The shaded area denotes the energy range within which states are occupied. *E*_CBM_corresponds to the energy level of the conduction band minimum.

Figure 4shows the calculated band structures of the polar La-doped BTO at various La concentrations. It is evident that as a result of tetragonal crystal field splitting (CFS), the Ti-*t*_2g_bands are split into two branches: one composed of *d*_xy_orbital and the other composed of *d*_xz,yz_orbitals (the energy separation between these branches is denoted by Δ_CFS_). The latter branch is split further into two sub-branches due to spin-orbit interactions (indicated by Δ_SOI_). In the polar structures, the CFS acts such that light mass *d*_xy_bands always fall energetically below heavy mass *d*_xz,yz_bands. The opposite situation is found for the non-polar state; light *d*_xy_bands lie above heavy bands. As a result, at low La concentrations, light (heavy) bands predominantly occupy the polar (non-polar) phase. As is shown in Fig. 4, by increasing *x*, the tetragonal Δ_CFS_of the polar phase dramatically decreases from 220 meV at *x* = 1% to 35 meV at *x* = 8.8%. This result accordingly leads to a destabilization of the ferroelectric phase and thus to a reduction of *T*_C_as observed experimentally. More importantly, due to the decrease in Δ_CFS_and increase in *x*, conducting electrons occupy both light *d*_xy_and heavy *d*_xz,yz_bands. In a thin film, the presence of heavy *d*_xz,yz_electrons can unfavourably affect electric transport and can result in a relative suppression of metallicity. This reasoning confirms our experimental finding that in La-doped BTO films, metallic behaviour is enhanced in the polar state rather than in the non-polar state. It is worth mentioning that by increasing *x*, the band structures of polar and non-polar phases become similar. For example, if we compare the band structure of polar La-doped BTO at *x* = 8.8% with the corresponding non-polar band structure, it is clear that in both cases, the Fermi level is almost at the same energy level and heavy bands accommodate most of the conducting electrons (see Fig. 4). Such a drastic change in the orbital character of conducting electrons in the polar phase is thus expected to be the main cause of abrupt *ρ*(*T*) slope changes around the transitional temperature at a low *x*(represented by the kink structure of the corresponding *ρ*(*T*) curves in Fig. 3a), whereas it shows almost no change with a sufficiently high *x*.

## Discussion

In conclusion, we fabricated La-doped BTO films on GdScO_3_substrates via MOMBE. The temperature dependences of the *c*-axis lattice constant and of SHG intensity indicate that the polar transition temperature of La-BTO decreases as La doping levels increase. With low doping at 0.64% (*n* = 1.0 × 10^20^ cm^−3^), the film exhibits metallic behaviour down to 100 K. The behaviour by which such metallicity is enhanced at the polar phase for low-doped films appears to be explained by our first-principle calculations, which reveal a characteristic change in the band structure across the phase transition of a low doping regime. We also presented a phase diagram of polar/non-polar and metal/insulator states. Although the destabilization of the polar phase by electron doping and stabilization by compressive strain compete against each other, we found that for a wide range of La concentrations, this strain effect wins and thus causes the system to remain in the polar metal phase. It is interesting that the unique polar metal phase is discovered via chemical carrier doping in BTO films, because such chemical doping is a typical method used to induce an insulator-metal transition in transition metal oxides, and various ferroelectric transition metal oxides can be fabricated by a superstructure^38^and via epitaxial strain^39^. This result suggests that carrier doping into a ferroelectric film stabilized by epitaxial strain can be used as an effective means to design and explore new polar metals.

## Methods

The La-BTO films were fabricated by MOMBE at a substrate temperature of 870 °C. The GSO substrates were annealed in air at 1,000 °C for 6 hours before the growth to form an atomically flat surface. Ba and La were generated from conventional effusion cells of each pure (Ba: 99.99% and La: 99.9%) metal source. Ba flux was maintained at a beam equivalent pressure (BEP) of 8 × 10^−8^ Torr. Titanium tetra-isopropoxide (TTIP) (99.9999%) as a Ti source was supplied via thermal evaporation (~100 °C) from a MO container bottle without any carrier gas. Distilled pure ozone gas was used as an oxidizing agent. We found that the direct growth of La-BTO films on GSO resulted in inferior crystalline quality but that the insertion of a 5 nm-thick BTO buffer layer dramatically improved crystalline quality. Therefore, all La-BTO films are composed of 5-nm-thick BTO buffer layer and of a 42–60-nm-thick La-doped BTO layer.

The films were characterized by optical second harmonic generation (SHG) with 1.55 eV fundamental photons (150 fs duration at a 1 kHz repetition rate) in the reflection geometry at a 45-degree incidence. Both incident and SH photons were *p*-polarized with the incident plane parallel to the (001)-plane of the GSO substrate. The signal was directed to colour filters and to a monochromator and was detected using a photomultiplier tube.

## Electronic supplementary material

Supplementary Information
