# Supplementary material for: Polar metal phase stabilized in strained La-doped BaTiO3films
Source: Sci Rep. 2017 Jul 5;7:4631. doi: 10.1038/s41598-017-04635-3 (PMC5498685; doi:10.1038/s41598-017-04635-3)
Supplement: Supplementary file 1 — Supplementary Information [file 41598_2017_4635_MOESM1_ESM.pdf]

Supplementary Information for

Polar metal phase stabilized in strained La-doped $BaTiO_3$ films

K. S. Takahashi, Y. Matsubara, M. S. Bahramy, N. Ogawa, D. Hashizume, Y. Tokura, and M. Kawasaki

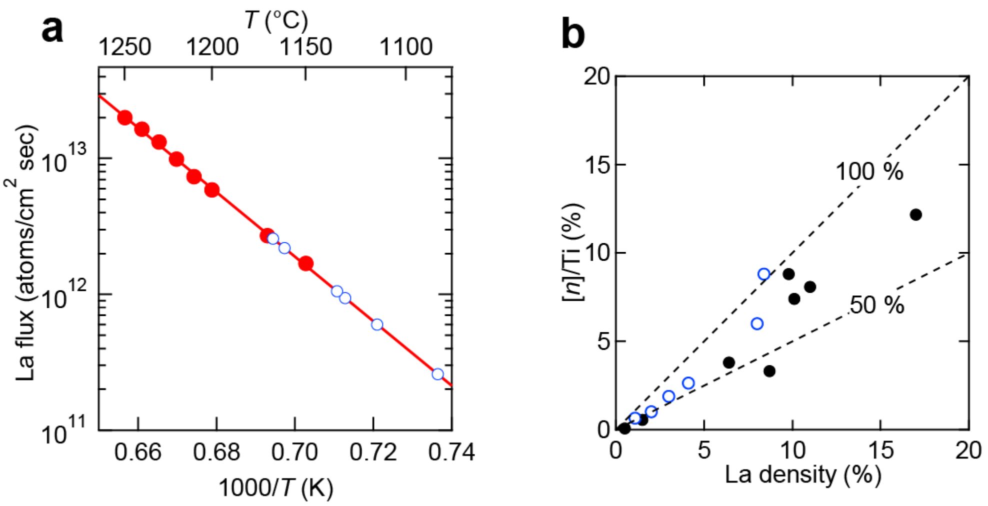

Supplementary Figure 1 (a) La flux determined by a quartz crystal microbalance is plotted as a function of 1000/*T* (red circles), which yielded in a linear fitting (red red line). The films in main text were grown at effusion cell temperatures denoted by open blue circles. (b) The relation between the carrier density [*n*]/Ti and La density for various La-BTO films. The [*n*]/Ti is determined by the Hall effect at 300 K and the La density is deduced by the fitting line of Supplementary Fig. 1(a). The films in main text are plotted as open blue circles while the others as solid black circles.

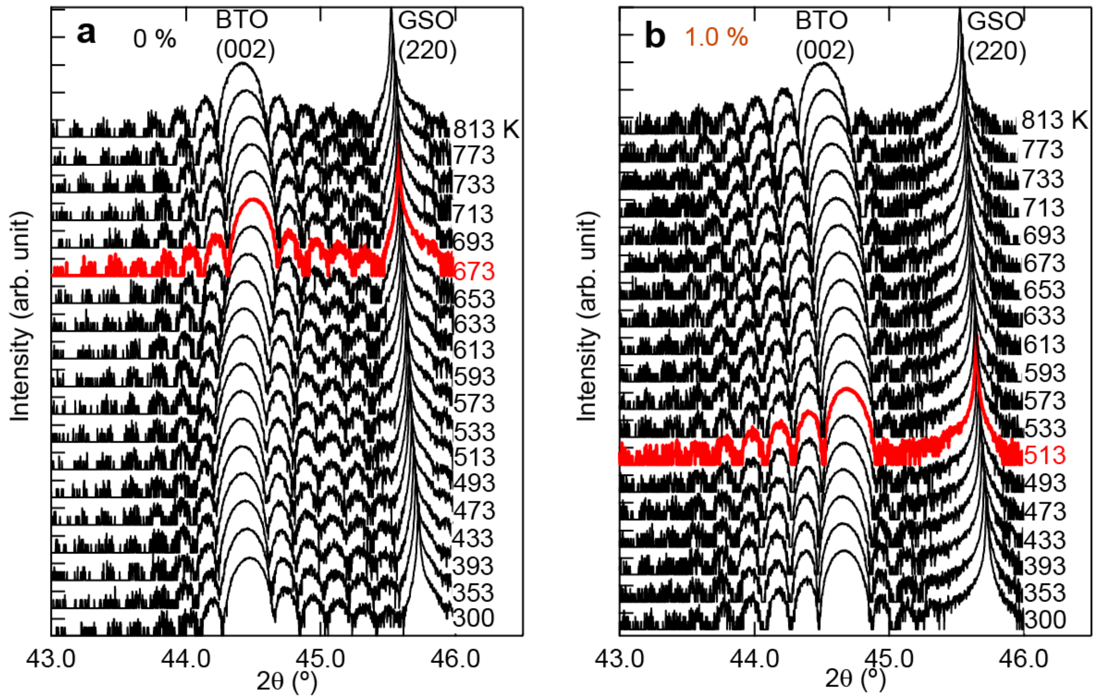

Supplementary Figure 2 2θ–θ x-ray diffraction patterns for the (002) peak of BTO and (220) peak of GSO at various temperatures for (a) non-doped BTO and (b) 1.0 % La doped BTO films. Red curves are the data at T_C.

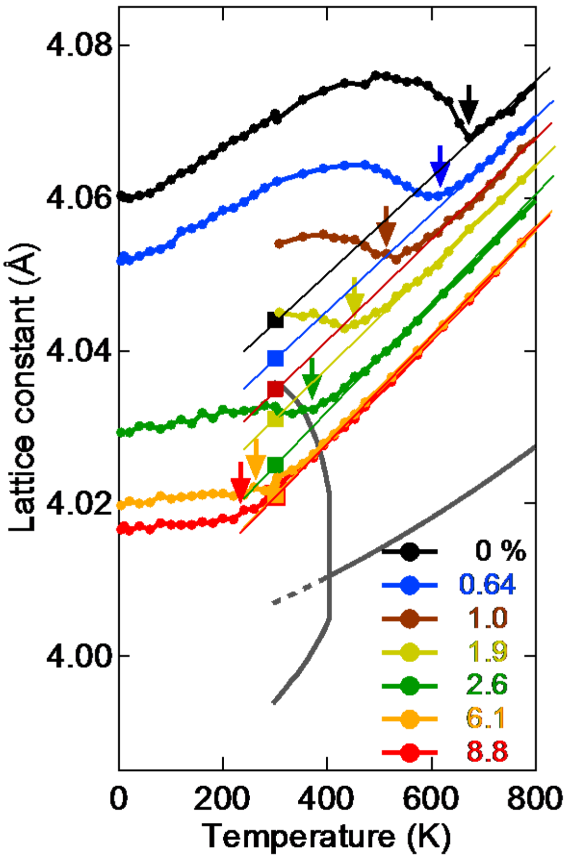

Supplementary Figure 3 Linear extrapolation of the *c*-axis lattice constant to room temperature for estimating c_{para} denoted as square symbols that is used to calculate $\overline{a}$ and ε for La-doped BTO films. For comparison, the temperature dependence of lattice constants of cubic and tetragonal phase of $BaTiO_3$ single crystal are also shown.

Supplementary Table 1 The lattice constants of c_{para} and $\overline{a}$ that are used to calculate ε for respective La-doped BTO films with various [n]/Ti.

**[n]/Ti (%)**	**0**	**0.64**	**1.0**	**1.9**	**2.6**	**6.1**	**8.8**
**c_{para} (Å)**	4.044	4.039	4.035	4.031	4.025	4.021	4.021
**$\overline{a}$ (Å)**	4.007	4.005	4.003	4.001	3.998	3.996	3.996
**ε (%)**	-0.92	-0.87	-0.82	-0.77	-0.70	-0.65	-0.65

Supplementary Note 1 | Carrier activation by La doping

In order to prescribe the La density in the films, we made a calibration curve between the La flux measured by a quartz crystal microbalance during MBE growth and La Knudsen cell temperature (*T*) as shown in Supplementary Fig. 1 (a). The red closed circles were measured values which can be well fitted with a linear line as Antoine equation. Because of the detection limit, the La flux for *T* lower than 1150°C cannot be measured. We set *T* to adjust lower La flux conditions according to the liner extrapolation as denoted by blue open circles to prepare the films discussed in main text. The actual carrier density determined by Hall effect measurement at room temperature is plotted in Supplementary Fig. 1 (b) as a function of the La density estimated by Supplementary Fig. 1 (a). The carrier activation ratio is between 50% and 100 %.

Supplementary Note 2 | Determination of in-plain lattice strain for La-doped BTO films.

To assess the relation between the T_C and the in-plane strain ε, we estimated the in-plane strain ε = (3.970 – $\overline{a}$)/$\overline{a}$. where $\overline{a}$ is the average lattice constant of the 'relaxed' paraelectric cubic phase and the value of 3.970 Å is the in-plane lattice constant of GSO(110) at room temperature. With $\overline{a}$ = 4.007 Å for BTO single crystal [1], the ε of non-doped BTO film on GSO is deduced to be –0.92 % (= (3.970 – 4.007)/4.007). To estimate the Poisson's ratio (ν) of a hypothetical non-doped BTO in paraelectric phase, the c-axis lattice constant of the strained film at room temperature on GSO (c_{para}) is extrapolated along the linear relation observed for the paraelectric phase above T_C as shown in Supplementary Fig. 3, yielding in 4.044 Å. By substituting the values in the equation, $\nu = \frac{c_{para} - \overline{a}}{(c_{para} - \overline{a}) - 2\times(3.970 - \overline{a})}$, ν = 0.33 is obtained. For La-doped BTO films, since the lattice constant $\overline{a}$ are not available, we have to estimate the $\overline{a}$ from experimental values to obtain ε. We estimated the $\overline{a}$ from the above equation, with assuming the same Poisson's ratio of 0.33 and extrapolated value c_{para} for respective La-BTO films. The results of c_{para}, $\overline{a}$, and ε are summarized in Supplementary Table 1. The ε values thus derived are plotted in Fig. 2c and d in main text.

Supplementary References

1. J. A. Bland The thermal expansion of cubic barium titanate ($BaTiO_3$) from 350 ºC to 1050 ºC, *Canadian Journal of Physics* **37** 417–421 (1959).
